# Microstructural Investigations of the Visual Pathways in Pediatric Epilepsy Neurosurgery: Insights From Multi-Shell Diffusion Magnetic Resonance Imaging

**DOI:** 10.3389/fnins.2020.00269

**Published:** 2020-04-08

**Authors:** Luís M. Lacerda, Jonathan D. Clayden, Sian E. Handley, Gavin P. Winston, Enrico Kaden, Martin Tisdall, J. Helen Cross, Alki Liasis, Chris A. Clark

**Affiliations:** ^1^Developmental Imaging and Biophysics Section, UCL Great Ormond Street Institute of Child Health, London, United Kingdom; ^2^Clinical and Academic Department of Ophthalmology, Great Ormond Street Hospital for Children NHS Foundation Trust, UCL Great Ormond Street Institute of Child Health, London, United Kingdom; ^3^Department of Clinical and Experimental Epilepsy, UCL Queen Square Institute of Neurology, London, United Kingdom; ^4^Division of Neurology, Department of Medicine, Queen’s University, Kingston, ON, Canada; ^5^Centre for Medical Image Computing, University College London, London, United Kingdom; ^6^Department of Neurosurgery, UCL Great Ormond Street Institute of Child Health, London, United Kingdom; ^7^Clinical Neurosciences, UCL Great Ormond Street Institute of Child Health, London, United Kingdom; ^8^Children’s Hospital of Pittsburgh, University of Pittsburgh Medical Centre, Pittsburgh, PA, United States

**Keywords:** pediatric, tractography, surgery, visual, clinical, multi-shell, diffusion

## Abstract

**Background:**

Surgery is a key approach for achieving seizure freedom in children with focal onset epilepsy. However, the resection can affect or be in the vicinity of the optic radiations. Multi-shell diffusion MRI and tractography can better characterize tissue structure and provide guidance to help minimize surgical related deficits. Whilst in adults tractography has been used to demonstrate that damage to the optic radiations leads to postoperative visual field deficits, this approach has yet to be properly explored in children.

**Objective:**

To demonstrate the capabilities of multi-shell diffusion MRI and tractography in characterizing microstructural changes in children with epilepsy pre- and post-surgery affecting the occipital, parietal or temporal lobes.

**Methods:**

Diffusion Tensor Imaging and the Spherical Mean Technique were used to investigate the microstructure of the optic radiations. Furthermore, tractography was used to evaluate whether pre-surgical reconstructions of the optic radiations overlap with the resection margin as measured using anatomical post-surgical T1-weighted MRI.

**Results:**

Increased diffusivity in patients compared to controls at baseline was observed with evidence of decreased diffusivity, anisotropy, and neurite orientation distribution in contralateral hemisphere after surgery. Pre-surgical optic radiation tractography overlapped with post-surgical resection margins in 20/43 (46%) children, and where visual data was available before and after surgery, the presence of overlap indicated a visual field deficit.

**Conclusion:**

This is the first report in a pediatric series which highlights the relevance of tractography for future pre-surgical evaluation in children undergoing epilepsy surgery and the usefulness of multi-shell diffusion MRI to characterize brain microstructure in these patients.

## Introduction

Resective surgery remains one of the most effective approaches to cure seizures in patients with drug resistant focal epilepsy (Ryvlin et al., 2014). In adults, the most common epilepsy surgery performed is anterior temporal lobe resection (ATLR), where the temporal pole, amygdala and anterior part of the hippocampus are removed (Kwan et al., 2011). Such surgery may damage the optic radiation (Winston et al., 2012). The optic radiations depart from the lateral geniculate nuclei (LGN) and subdivide into three main bundles: a posterior bundle and central bundle that run straight back to the occipital lobe and an anterior bundle, that runs anteriorly alongside the lateral ventricle, projecting deep within the temporal lobe before turning backward at the level of the temporal horn to join the first two bundles (Ebeling and Reulen, 1988; Rubino et al., 2005). The anterior bundle, most often denominated Meyer’s Loop, is most at risk in the types of resections described above, and the correlation between the extent of its resection and resulting visual field deficits following surgery in adults is well established (Falconer and Wilson, 1958). Diffusion MRI allows extraction of measures that identify the main directions of water molecular displacement and from that a three-dimensional trajectory of fiber bundles can be reconstructed [tractography (Conturo et al., 1999) and has been used in numerous studies to reconstruct the optic radiations (Yogarajah et al., 2009; Clatworthy et al., 2010; Martínez-Heras et al., 2015; Chamberland et al., 2017)]. Despite the success of tractography in demonstrating involvement of the optic radiations in the resected tissue and subsequent correlation between the extent of optic radiation involvement and post-surgical visual fields in adults (Yam et al., 2010; Winston et al., 2011, 2012, 2014a), there is very little literature on the use of the technique in pediatric series. Addressing this issue is clearly an important step to determine the relevance of optic radiation tractography as a potential neurosurgical planning tool in children undergoing surgery for intractable epilepsy, where there is a greater variety of surgical approaches reflecting the broader and more diverse pathological and neuro-anatomical involvement. As such, surgical damage to the visual system may be more varied than in adults resulting in a wider variety of visual function disturbance than visual field deficits alone (Lee et al., 2002). Optic radiation tractography in healthy children has been well-documented in our previous work, including anatomical distances that are relevant to epilepsy surgery (Dayan et al., 2015b). Furthermore, based on studies in healthy adults, we have demonstrated optic radiation tractography to be robust and reproducible (Dayan et al., 2015a, b). Diffusion tensor imaging (DTI) (Basser et al., 1994) has been applied successfully in clinical practice but associated metrics such as fractional anisotropy are sensitive to numerous tissue properties including axonal density, diameter and orientation (Alexander et al., 2007; Jones et al., 2013). Building on the knowledge provided by DTI, more advanced diffusion signal modeling methods have been developed in recent years to explore the microscopic environment in clinically feasible scan times (Zhang et al., 2012; Farquharson et al., 2013; Lasič et al., 2014; Kaden et al., 2016a; Salama et al., 2018). This has been made possible due to developments in acquisition technology, such as multi-band echo planar imaging (Setsompop et al., 2012a, b) and multi-shell diffusion MRI in which a second shell or *b*-value of diffusion sensitizing directions is added to the initial single shell required for DTI. Here we focus on the spherical means technique (SMT), which is based on the observation that for any fixed *b*-value, the spherical mean of the diffusion signal over the gradient directions does not depend on the orientational structure (Kaden et al., 2016b). This insight permits disentangling microstructural tissue features from orientational effects such as fiber crossings and orientation dispersion that confound traditional DTI metrics.

## Main Objectives

The aim of the present study was to utilize multi-shell diffusion MRI and tractography to delineate the posterior part of the visual pathway in children who have undergone epilepsy surgery involving the occipital, parietal and temporal lobes to:

1. Determine the frequency and location of optic radiation involvement relative to the resection and to relate these to information regarding measures of visual function where clinically available.
2. Investigate pre-existing differences in tissue microstructure between healthy subjects and the patient cohort before surgery and determine differences between the hemisphere where the operation would take place (“ipsilateral”), in comparison with the healthy hemisphere (“contralateral”).
3. Investigate the effects of surgery on the contralateral hemisphere by evaluating differences between the pre- and post-surgical optic radiation microstructure.

## Materials and Methods

### Ethics and Patient Information

This manuscript results from a retrospective study registered as a Case Note Review, with access to previously collected, non-identifiable information/data under GAfREC 2011 and as a result is exempt from REC approval and from patient consent. An approval was issued by the relevant Trust for the study to proceed. All patients were medically refractory to anti-epileptic drugs as per the ILAE definition (Téllez-Zenteno et al., 2014). Patients that had undergone resective epilepsy surgery affecting the temporal, parietal and occipital lobes and that may therefore have involved the optic radiations were selected for this study (age at operation 5–19 years). Patients undergoing hemispherectomy were excluded. In total 43 patients with median age of 10.70 years (Inter-quartile range 8.4; 22 male) were included in the study. Information about seizure freedom at >1 year was available for 36/43 patients and indicated a seizure freedom rate of 75%. All information about demographics, type of operation performed, medication and times of scans in relation to the surgery can be found in the Table 1.

**TABLE 1:** Detailed clinical information for selected patient population.

**Patient**	**Age at surgery (years)**	**Scan after surgery (days)**	**Visual assessment after surgery (days)**	**Gender**	**Surgery**	**Seizure freedom > 1 year**	**Medication**
Patient01	16.0	179	No data	F	L temporal lesionectomy	Yes	Keppra
Patient02	7.0	137	No data	M	L anterior temporal lobectomy	No	Phenytoin, sodium valproate and zonisamide
Patient03	7.0	236	No data	M	L anterior temporal lobectomy and amygdalo-hippocampectomy	Yes	Sodium valproate, lamotrigine
Patient04	13.0	100	267	F	L anterior temporal lobectomy and amygdalo-hippocampectomy	Yes	Oxcarbazepine, zonisamide
Patient05	15.0	167	No data	F	L anterior temporal lobectomy and amygdalo-hippocampectomy	Yes	Carbamazepine, sodium valproate, clobazam
Patient06	6.0	559	No data	F	L frontal, anterior temporal lobectomy and amygdalectomy	No	Lacosamide, phenobarbitone
Patient07	16.0	200	No data	M	L posterior temporal lesionectomy	Yes	Oxcarbazepine, sodim valproate, levetiracetam
Patient08	12.0	168	No data	M	L posterior temporal lesionectomy	Yes	Carbamazepine, clonazepam
Patient09	17.0	82	140	F	L temporal lesionectomy	Yes	Tegretol
Patient10	6.0	117	No data	M	L temporal lesionectomy	Yes	Levetiracetam, clobazam
Patient11	7.0	286	No data	F	L anterior temporal lesionectomy and amygdalo-hippocampectomy	No	Topiramate, clobazam
Patient12	18.0	102	320	M	L temporal lobectomy (extended)	No	Lamotrigine, lacosamide
Patient13	11.0	4	50	M	L temporal lobectomy and amygdalo-hippocampectomy (extended)	Yes	Carbamazepine, sodium valproate, levetiracetam
Patient14	12.0	158	161	M	L temporo-occipito-parietal disconnection	Yes	Perampanel, sodium valproate, levetiracetam
Patient15	12.0	89	98	M	L temporo-occipito-parietal disconnection	Yes	Carbamazepine, topiramate
Patient16	11.0	165	No data	M	L temporo-occipito-parietal disconnection	Yes	Carbamazepine, clobazam
Patient17	5.0	137	54	M	R fronto-temporo-parietal resection	Yes	Levetiracetam, clobazam
Patient18	14.0	81	64	F	R occipital lesionectomy	Yes	Sodium valproate, levetiracetam
Patient19	7.0	186	No data	F	R occipital lesionectomy	No	Sodium valproate, levetiracetam
Patient20	10.0	308	No data	F	R anterior temporal lobectomy and amygdalo-hippocampectomy	Yes	Sodium valproate, vigabatrin
Patient21	19.0	237	No data	F	R anterior temporal lobectomy	Yes	Oxcarbazepine
Patient22	12.0	411	264	M	R anterior temporal lobectomy and amygdalo-hippocampectomy	No	Oxcarbazepine, clobazam
Patient23	17.0	176	No data	M	R anterior temporal lobectomy and amygdalo-hippocampectomy	Yes	Levetiracetam
Patient24	7.0	160	No data	F	R anterior temporal lobectomy and amygdalo-hippocampectomy	No	Sodium valproate, lamotrigine, midazolam
Patient25	13.0	108	286	M	R temporal lesionectomy	Yes	–
Patient26	10.0	188	No data	F	R temporal lesionectomy	Yes	Levetiracetam
Patient27	6.0	287	No data	F	L temporal lesionectomy	Yes	Sodium valproate, lamotrigine
Patient28	11.0	242	No data	M	L parietal tumor resection	Yes	Zonisamide
Patient29	6.0	259	No data	F	R anterior temporal lesionectomy and amygdalo-hippocampectomy	Yes	Sodium valproate, topiramate
Patient30	6.0	154	No data	M	L temporal lobectomy	No	Topiramate, keppra
Patient31	5.0	176	111	F	R temporal lesionectomy	Yes	Keppra
Patient32	9.0	No data	333	M	L temporal lobectomy lesionectomy	No	Lacosamide, levetiracetam, lamotrigine, risperidone, phenytoin
Patient33	10.0	85	No data	F	L parietal lesionectomy	No	Sodium valproate, levetiracetam
Patient34	6.0	No data	No data	F	R occipital lobectomy	Yes	Keppra, lamotrigine
Patient35	13.0	89	No data	F	L temporal lobectomy and amygdalo-hippocampectomy	Yes	Carbamazepine
Patient36	8.0	No data	No data	M	R temporal lobectomy	No	Oxcarbazepine, lacosamide
Patient37	8.0	81	No data	M	L parietal lesionectomy	Unavailable	Sodium valproate, oxcarbazepine
Patient38	17.0	73	No data	F	R temporal lesionectomy and amygdalo-hippocampectomy	Unavailable	Keppra, oxcarbazepine, clobazam
Patient39	16.0	155	134	M	L temporo-occipito-parietal disconnection	Unavailable	Carbamazepine, lacosamide, phenobarbitone
Patient40	18.0	No data	No data	F	L temporal lesionectomy	Unavailable	Oxcarbazepine, midazolam
Patient41	18.0	No data	No data	F	L parietal lesionectomy	Unavailable	Topiramate, lamotrigine, levothyroxine
Patient42	5.0	125	239	M	R temporo-occipito-parietal disconnection	Unavailable	Carbamazepine
Patient43	15.0	102	299	F	L temporo-occipito-parietal disconnection	Unavailable	Lacosamide, levetiracetam, lamotrigine

*R, right; L, left; F, female; M, male.*

**FIGURE 1:**
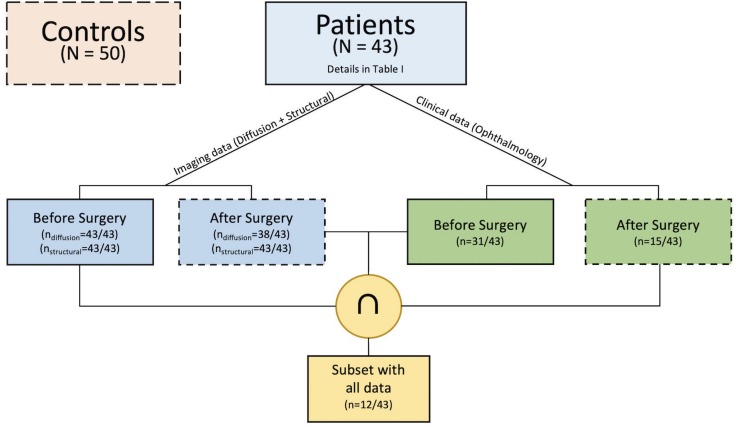
Structure of the data available for the current study.

Pre-operatively structural T1-weighted and multi-shell diffusion MRI data were available for all patients whilst pre-surgical ophthalmological examination was available for 31 patients. Post-operative structural and multi-shell diffusion MRI were available for all and 38 patients, respectively, whilst follow-up ophthalmological examination was available for 15 patients (Figure 1). Of the entire cohort there were 12 patients that had complete sets of data. Additionally, imaging data from a cohort of 50 healthy children and young adults with no visual or neurological conditions and median age of 11 years (Inter-quartile range 6.5; 30 male) was also selected to evaluate possible differences in optic radiation microstructure when compared to the patient group; further details are provided in the main objectives section. Patient and control groups are both age (*F* = 1.31, df = 91, *p*-value = 0.2556) and gender (χ^2^ = 0.73243, df = 1, *p*-value = 0.3921) matched.

### Imaging Data

All imaging data were acquired on a Siemens 3.0 T Prisma scanner (Siemens, Erlangen, Germany), equipped with a 20-channel head receive coil. The protocol included a multi-shell diffusion sequence employing a diffusion-weighted spin-echo single shot 2D EPI acquisition, with multi-band radio frequency pulses to accelerate volume coverage along the slice direction (Setsompop et al., 2012a, b; Auerbach et al., 2013). A multi-band factor of 2 was used to image 66 slices of 2 mm thickness with 0.2 mm slice gap. Diffusion gradients were applied over two ‘shells’: *b* = 1000 s/mm^2^ and *b* = 2200 s/mm^2^, with 60 non-collinear diffusion directions per shell in addition to 13 interleaved *b* = 0 (non-diffusion weighted) images. Other imaging parameters were: TR = 3050 ms, TE = 60 ms, field of view = 220 mm × 220 mm, matrix size = 110 × 110, in-plane voxel resolution = 2.0 mm × 2.0 mm, GRAPPA factor 2, phase-encoding (PE) partial Fourier = 6/8. An additional *b* = 0 acquisition was performed, with identical readout to the diffusion-weighted scan, but with the phase encode direction flipped by 180° (in the anterior-posterior direction), for correction of susceptibility-related artifacts. The total scan time for the multi-shell diffusion sequence (including the *b* = 0 acquisition with flipped PE) was 7 min 50 s. In addition, a T1-weighted MPRAGE structural image was acquired using 176 contiguous sagittal slices, FOV = 256 mm × 240 mm and 1 × 1 × 1mm image resolution; TE = 4.9 ms and TR = 11 ms.

### Pre-processing and Reconstruction of Maps

Multi-shell diffusion MRI data were denoised using MRtrix3’s implementation of the method developed by Veraart et al. (2016a, b). Furthermore, FSL (Jenkinson et al., 2012) commands TOPUP and EDDY were used to correct for susceptibility distortions and to perform motion and eddy current correction (Andersson and Sotiropoulos, 2016). After pre-processing, multi-shell diffusion MRI data was used in the MRtrix3 Constrained Spherical Deconvolution (CSD) pipeline, estimating both the multi-tissue response function and fiber orientation distribution (FOD) fields (Tournier et al., 2012). Freesurfer was used for bias field correction and skull stripping of pre-surgical structural T1-weighted scans and to provide parcelation of cortical and subcortical structures based on a standard atlas (Fischl, 2012). As Freesurfer was developed mainly for adult brains, in this study, regions from the derived cortical and subcortical parcelation, namely the thalamus and occipital cortices, were visually inspected and edited when required for each subject, before being used as regions of interest for performing optic radiation tractography. DTI maps were reconstructed with MRtrix3 – fractional anisotropy (FA), mean diffusivity (MD), axial diffusivity (AD), and radial diffusivity (RD). The SMT microscopic tensor was also fit and provided estimates of the microscopic diffusivities parallel (Long) and perpendicular to the neurites (Trans) from which the Microscopic fractional anisotropy (μFA) and Microscopic mean diffusivity (μMD) were calculated (Kaden et al., 2016b). Further to the measurement of μFA which reflects the degree of directionality arising from the local diffusion processes, the SMT technique also enables the reconstruction of the Orientation Dispersion Entropy (ODEntropy) reflecting the microdomain orientation distribution, taking higher values for regions constituted by highly coherent bundles and a lower value for more uniformly distributed substrates such as gray matter or the ventricular system.

### Tractography Reconstructions

The Freesurfer parcelations were registered to diffusion space by registering the structural skull stripped data to the mean *b* = 0 image calculated for each subject, following a rigid body transformation and non-linear deformations applied with ANTS (Avants et al., 2014). Seeding and inclusion regions of interest, comprising the thalamus and a composite occipital region – consisting of lingual, pericalcarine, lateral occipital cortical regions – were generated for each subject. In order to determine the starting point of the optic radiation tractography – i.e., the LGN – the center of mass of the thalamus region was calculated and used to divide it in two halves; the posterior half of the thalamic region was then retained as the seed region. Additional regions of interest to exclude artifactual contributions were extracted from the Freesurfer parcelation, namely the contralateral gray matter mask. Finally sagittal, axial and coronal ROIs were drawn for each individual subject in order to exclude lateral projections from the acoustic radiation, posterior projections of the corpus callosum, parts of the cingulum bundle and brainstem (Dayan et al., 2015a, b; Kammen et al., 2016; Nowell et al., 2016; Chamberland et al., 2017). These regions were used to perform tractography with a probabilistic algorithm in MRTrix3 with 2nd order integration over the fiber orientation distribution [iFOD2 (Tournier et al., 2010)] with a 90° angular threshold, 5000 selected streamlines and FA and FOD amplitude threshold of 0.15 and 0.1, respectively. The tractography were then multiplied by the individual DTI and SMT maps, and a weighted average of each metric calculated, resulting in single value for each metric within the resulting optic radiation region.

### Resection Segmentation and Pre and Post-surgical MRI Registration

**FIGURE 2:**
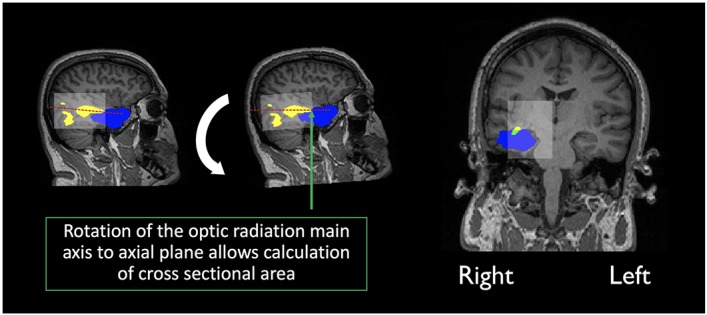
Calculation of the overlap score: Yellow indicates the optic radiation reconstruction, Blue the resection area and green the overlap measure, calculated as the percentage of the optic radiation disconnection, at maximum cross-sectional area. In transparent white there is a bounding box created by the limits of the optic radiation maps.

In order to assess overlap between the surgical resection and tractography prior to surgery, the pre-surgical reconstructions were first registered into post-surgical space. The first step consisted of semi-automatic segmentation of the resection, from the post-operative structural scan, using ITK-snap (Avants et al., 2014). The resulting resection mask was then visually inspected by a neurosurgeon (MT) and edited as required, to cover the entire extent of the resection. Following resection segmentation, the registration between pre and post-surgical scans was performed with the ANTS software (Tustison and Avants, 2013), selecting the derived segmentation as an exclusion mask in the registration procedure. The final step involved registering the pre-surgical tractography to post-surgical space. In order to achieve this, the original transformation files (from pre-structural to pre-diffusion space) were applied together with those derived in the previous step. Finally, the main axis along which the optic radiations maps (thresholded at 5%) are oriented was determined so that the optic radiations could be rotated to the axial plane (the same rotation was applied to the post-surgical structural volume and resected mask). The cross-sectional area was then calculated as the fraction of optic radiation voxels in common with the resected area over the whole optic radiation volume, at the coronal slice where overlap between both was maximum (Figure 2).

### Clinical Evaluation of Visual Pathways

The patient’s notes were retrospectively reviewed for pre- and post-operative ophthalmological examination undertaken, and specifically for documented visual field assessment. Visual fields were either tested by Goldmann perimetry or by confrontation methods using the two-examiner technique depending on the child’s age and ability at the assessment (Heidary, 2016). As the patients in this study underwent surgery as part of a national service, pre and/or post-surgery ophthalmology has on occasion been performed by local services and as a result visual function data pre- and post-operatively is not available for the some of subjects in the selected cohort (further discussion in the limitations section). Pre-operatively 31 patients had visual field assessment and 2 were abandoned due to poor co-operation, 26 were normal and 3 were abnormal. Post-operatively 15 had visual field assessment out of which 2 were abandoned due to poor co-operation, 5 were normal and 8 were abnormal. Of the 12 patients who had pre- and post-operative testing, 5 were normal throughout, 1 had a deficit pre and post-operatively, the remaining 6 had acquired visual field deficits. Although the ophthalmological data in this report is limited, it was sufficient to establish the degree of visual field deficit (normal, quadrantanopia, or hemianopia) using appropriate methods.

### Statistical Analyses

After extraction of all metrics a linear mixed-effects model was fit to the data in R (*lme4* library in R^1^) with age, gender, group (patient/control) and timepoint (baseline/after surgery) as fixed effects, and a random effect of subject-specific intercepts and hemisphere. Hemisphere was encoded as ipsilateral vs. contralateral in patients, and left vs. right differences in controls, as to account for the variability across group. The package *lmerTest* was then used to evaluate significant effects found with the fitted model the results of which can be found in Table 2.

**TABLE 2:** Mixed effect model results.

	**Effect**	**Estimate**	**Std. Error**	***t*-value**		**Effect**	**Estimate**	**Std. Error**	***t*-value**
MD	Intercept	7.94E-04	8.04E-06	98.753***	μMD	Intercept	1.07E-03	8.53E-06	125.31***
	Group	3.01E-05	5.01E-06	−6.011***		Group	3.98E-05	5.40E-06	−7.372***
	Timepoint	−5.71E-07	2.46E-06	−0.232		Timepoint	−6.29E-06	3.32E-06	−1.896
	Gender	1.48E-05	4.92E-06	2.999**		Gender	1.62E-05	5.20E-06	3.119**
	Age	−5.29E-06	6.12E-07	−8.651***		Age	−6.04E-06	6.47E-07	−9.335***
AD	Intercept	1.23E-03	9.38E-06	130.786***	Long	Intercept	2.68E-03	1.72E-05	155.745***
	Group	4.13E-05	5.93E-06	−6.965***		Group	7.96E-05	1.09E-05	−7.307***
	Timepoint	−3.95E-05	4.34E-06	−9.109***		Timepoint	−4.12E-05	7.70E-06	−5.343***
	Gender	2.40E-05	5.72E-06	4.187***		Gender	3.38E-05	1.05E-05	3.213**
	Age	−4.18E-06	7.12E-07	−5.868***		Age	−9.39E-06	1.31E-06	−7.182***
RD	Intercept	5.81E-04	9.56E-06	60.733***	Trans	Intercept	2.61E-04	7.53E-06	34.687***
	Group	2.74E-05	5.97E-06	−4.595***		Group	1.97E-05	4.68E-06	−4.217***
	Timepoint	1.65E-05	3.15E-06	5.227***		Timepoint	1.03E-05	2.28E-06	4.53***
	Gender	1.21E-05	5.84E-06	2.067*		Gender	9.82E-06	4.61E-06	2.132*
	Age	−5.92E-06	7.27E-07	−8.146***		Age	−4.27E-06	5.73E-07	−7.446***
FA	Intercept	4.32E-01	7.68E-03	56.281***	μFA	Intercept	8.86E-01	3.36E-03	263.751***
	Group	-8.37E-03	4.82E-03	1.737		Group	−4.86E-03	2.10E-03	2.319*
	Timepoint	−2.80E-02	3.16E-03	−8.885***		Timepoint	−6.58E-03	1.20E-03	−5.494***
	Gender	−1.34E-03	4.69E-03	−0.286		Gender	−3.00E-03	2.05E-03	−1.459
	Age	3.76E-03	5.84E-04	6.439***		Age	1.66E-03	2.56E-04	6.506***
					OD Entropy	Intercept	4.65E-01	1.12E-02	41.618***
						Group	−1.07E-02	6.89E-03	1.552
						Timepoint	−4.37E-02	4.92E-03	−8.887***
						Gender	5.16E-03	6.85E-03	0.753
						Age	4.50E-03	8.54E-04	5.274***

Fixed effects and their significance are presented for the different metrics extracted within the optic radiations according to the following legend: *p < 0.05, **p < 0.01, and ***p < 0.001.

## Results

Whilst age and gender displayed several significant associations, we were mostly interested in exploring the effect of surgery as well as group and so report on these: Pre-surgically, both DTI and SMT revealed significant increased diffusivity metrics in patients compared to controls, with further significant anisotropy decrease detected by SMT, while there were no differences in the neurite orientation dispersion as measured by ODEntropy. After surgery, we observed a decrease in contralateral hemisphere AD, FA, Long, μFA, and ODEntropy while RD and Trans were increased in comparison to the pre-surgical optic radiations (a summary of these changes can be found in Table 3).

**TABLE 3:** Summary of significant increases (↑) and decreases (↓) found as a result of the fixed effects of the fitted model, both before and after surgery.

	**Patients vs. controls (at baseline)**	**Pre-surgical contralateral vs. Post-surgical contralateral hemisphere (patients)**
**DTI**		
MD	↑	–
AD	↑	↓
RD	↑	↑
FA	–	↓
**SMT**		
μMD	↑	–
Long	↑	↓
Trans	↑	↑
μFA	↓	↓
ODEntropy	–	↓

*Trend of non-significant changes (–) can be further investigated in Table 2. DTI, diffusion tensor imaging metrics; FA, fractional anisotropy; MD, mean diffusivity; AD, axial diffusivity; RD, radial diffusivity; SMT metrics, spherical mean technique; Long, parallel microscopic diffusivity; Trans, perpendicular microscopic diffusivity; μFA, microscopic fractional anisotropy; μMD, Microscopic mean diffusivity.*

**FIGURE 3:**
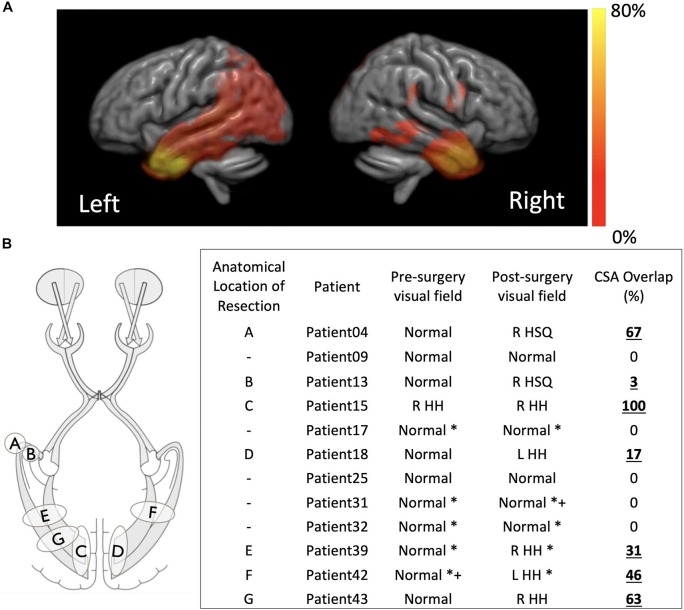
**(A)** Distribution of the resection margins for the 43 subjects in this study, per hemisphere, overlapped onto a template generated by registering all the individual structural scans. Hot color scale indicates the percentage of the total operations that were performed in a specific location. The maximum of the hot color scale indicates the highest incidence of surgical procedures for both hemispheres. **(B)** For a smaller subset of patients for which visual function data was available before and after surgery a small sketch of the resection locations and a table with overlap between pre-surgical tractography of the optic radiations and post-surgical structural scan are presented. R, right; L, left; HH, homonymous hemianopia; HSQ, homonymous superior quadrantanopia. *confrontational, +supported with electrophysiological data.

In 20/43 children there was overlap between the pre-surgical optic radiation tractography and post-surgical resection margin on T1-weighted MRI as measured by tractography. Where visual field data were available before and after surgery the presence of resection overlap with tractography corresponded to abnormal post-surgical visual function following normal pre-surgical visual function in every instance – patients 4, 13, 15, 18, 39, 42, and 43 (Figure 3). In patients with no pre-existing or post-surgical deficits no corresponding overlap of the optic radiations with the resection margin was found – patients 9, 17, 25, 31, and 32 (Figure 3). The overlap scores for the remaining patients can be found in the Supplementary Table 1.

## Discussion

Advanced multi-shell diffusion imaging and tractography was used to detect changes in the structure of the visual pathways in a cohort of children undergoing epilepsy surgery involving the occipital, parietal, and temporal lobes. Additionally, tractography was used to determine optic radiation involvement in the resection margin and where follow-up ophthalmological data was available to determine the correspondence between these.

### Investigation of Microstructural Properties of Optic Radiations

There were no significant differences in the microstructural properties of the optic radiations at baseline between the contralateral and ipsilateral sides in patients, nor between left and right hemisphere in controls (i.e., random effects were centered around zero – data not shown). Comparisons between patients and controls revealed statistically significant increases in MD, AD and RD which is consistent with previous findings in adults (Winston et al., 2014b; Bonilha et al., 2015). Further, anisotropy decreases in patients compared to controls were also found although only reaching significance as probed with μFA (from SMT) and not FA. SMT does not only provide metrics of tissue microstructure not confounded by orientational effects, but also estimates the orientation dispersion entropy, a measure of the neurite orientation distribution which was comparable between patients and controls. Remaining SMT parameters revealed significant increases in longitudinal, mean, and transverse microscopic diffusivities. These results are very likely explained by the increase in extracellular fluid that is observed in epilepsy, as well as altered myelination patterns and axonal loss (Concha et al., 2005). When comparing the contralateral hemisphere before and after surgery statistically significant decreases in AD, FA, Long, μFA, and OD Entropy as well as an increase in RD and transverse microscopic diffusivity were found. Taken together these findings indicate changes to the microstructural organization of the white matter with greater dispersion of structures and microdomains being less anisotropic. It is unclear exactly what processes relate to these changes although it is possible that they represent a process of re-organization. It has been documented previously that as a result of surgery there is some reorganization that takes place in the brain (Taylor et al., 2018), and that it is generally associated to increased anisotropy in the contralateral hemisphere (Yogarajah et al., 2010; Winston et al., 2014b). However, a recent study (Li et al., 2019) shows that this trend is more prominent after 6 months following surgery, following an initial decrease in anisotropy documented at 3 months post-surgery in the contralateral optic radiation. The pediatric cohort studied here had varied intervals for follow-up imaging limiting the conclusions that can be drawn regarding the timescale of possible re-organization and its effect on diffusion metrics. Future work should ideally obtain several follow-up scans at fixed time points for all subjects in order to determine unambiguously the evolution of diffusion parameters post-surgically.

### Clinical Correlations With Tractography

Despite the high anatomical variability and marked asymmetry that is associated with the distance of Meyer’s Loop to the temporal pole between hemispheres (Bürgel et al., 2006; Jeelani et al., 2010; Dreessen de Gervai et al., 2014), in this study we were able to successfully map the optic radiation in all subjects. In particular, we were able to do so in a pediatric surgical cohort and show, to the best of our knowledge, the first report that relates the degree and location of resection extent with optic radiation involvement in a pediatric surgical series. Although it is also self-evident that disruption of optic pathways is related to postoperative visual field deficit, the location of the resection and the nature of the developed visual field deficit have not been well explored (Kivelev et al., 2012). Furthermore, whereas in adults there have been many studies linking the impact of surgery on brain function, in particular memory (Duncan, 2008), this has yet to be addressed in pediatric cohorts (Berg, 2015), where the incidence of neurological complications is higher than in adults (Hader et al., 2013). One study in particular reported a higher rate of major visual field deficits in children when compared to adults (38% vs. 14%) and a lower rate of minor visual field deficits in children when compared to adults (6.35 vs. 16%). The higher variability in estimates of the rate of visual field deficits following epilepsy surgery in a pediatric cohort in comparison to previous adult reports, likely arises due to the more diverse surgeries performed as opposed to pure anterior temporal lobectomies that are performed in adults (Hader et al., 2013; Schmeiser et al., 2017) and due to the ability of the brain to reorganize and revert to some extent the severity of visual field deficit initially determined (Yam et al., 2010). In this study, the optic radiations were found to be involved in the resection in 20/43 (46%) patients. Where both pre- and post-surgical ophthalmological data was available, 7/12 (58%) had a demonstrable visual field deficit, corresponding to the presence of a cross-sectional area (CSA) overlap of the resection with the pre-surgical tractography (see Figure 3). Whilst typically the type of analysis performed in adults when identifying relationships between visual pathways and post-surgical deficits focus on the Meyer’s loop – temporal pole distance (Yogarajah et al., 2009; Mandelstam, 2012; Lilja et al., 2015), in children the variability in surgery across patients which could affect any part of the optic radiation demands a different approach, hence here the cross-sectional overlap between the optic radiation and the resection volume was determined.

### Limitations and Future Directions

Whilst surgery still remains the most effective course of action to drug resistant epilepsy (Schmidt and Stavem, 2009; Jobst and Cascino, 2015), it is well known that the brain shifts position during surgery, and this has been addressed using tractography during neurosurgical procedures (Nimsky et al., 2005; Winston et al., 2012; Yang et al., 2017). It has also been documented that there is degeneration of the visual system over time following damage to post-chiasmal visual pathways (Millington et al., 2014). In some cases, the long-time interval between the pre-surgical scan and surgery may have resulted in changes in the position of the visual pathways by the time surgery was performed, as well as in other brain regions reflecting brain plasticity (Yogarajah et al., 2010). Furthermore, it is also possible, that following surgery there may have been some morphological changes over time, namely, the occupation of the resection cavity by the remaining brain tissue. The timeframe of these changes is not clear, but if this is the case, it is possible that brain shift has contributed to an underestimation of the segmented resection area. In the absence of proper a validation technique other than post-mortem dissections, the comparison between pre- and intra-operative tractography reconstructions as well as the combination with other MRI modalities and electrophysiological recordings should also provide further assurance regarding the use of tractography for neurosurgical purposes (Schmitt et al., 2014; Alvarez et al., 2015; Nooij et al., 2015; Nimsky et al., 2016). A further limitation is the absence of a complete set of ophthalmology data in all the patients studied, although in the data available a clear correspondence between optic radiation involvement and visual field defect was demonstrated. Finally, SMT is able to provide an alternative to explore microstructural changes following surgery in epilepsy when compared to traditional DTI parameters. The technique is still relatively new but does provide unambiguous assessments of tissue dispersion and microscopic anisotropy both of which were found to have changed in the post-surgical optic radiations indicating a potential re-organization phenomenon. Further work is required however, to fully determine the time evolution and significance of these observations.

## Conclusion

In this study tractography reconstructions of the optic radiations were successfully performed in a pediatric cohort undergoing epilepsy surgery and used to evaluate the overlap of pre-surgical reconstructions with the resected area after surgery. In a cohort of 43 children the optic radiations were involved in the resection margin in 46% of cases. Of those with follow-up ophthalmology data all of the 7 patients with a demonstrable overlap of the optic radiations with the resection volume had a visual field deficit whereas the remaining patients with no involvement of the optic radiations in the resection margin had no evidence of a visual field deficit. Microstructural differences of the optic radiations in patients with epilepsy when compared with controls were observed with higher diffusivity metrics and lower anisotropy, with a comparable (neurite) orientation distribution as measured by SMT. Evidence of changes in the contralateral hemisphere following surgery, namely decreases in anisotropy and (neurite) orientation distribution accompanied by increased diffusivity in the radial direction were also found and are possibly related to a process of microstructural re-organization. Finally, this is the first report in a pediatric series that highlights the relevance and importance of multi-shell diffusion MRI and optic radiation tractography for pre-surgical evaluation in children undergoing epilepsy surgery affecting the occipital, parietal or temporal lobes.

## Data Availability Statement

The datasets generated for this study are available on request to the corresponding author.

## Ethics Statement

Ethical review and approval was not required for the study on human participants in accordance with the local legislation and institutional requirements. Written informed consent to participate in this study was provided by the participants’ legal guardian/next of kin.

## Author Contributions

LL, SH, JC, and CC contributed to conception and design of the study. JC performed the statistical analysis. LL wrote the first draft of the manuscript. All authors contributed to manuscript revision, read and approved the submitted version.

## Conflict of Interest

The authors declare that the research was conducted in the absence of any commercial or financial relationships that could be construed as a potential conflict of interest.

The handling Editor declared a past co-authorship with one of the authors EK.

## Footnotes

1 www.r-project.org
