# Supplementary material for: Microstructural Investigations of the Visual Pathways in Pediatric Epilepsy Neurosurgery: Insights From Multi-Shell Diffusion Magnetic Resonance Imaging
Source: Front Neurosci. 2020 Apr 8;14:269. doi: 10.3389/fnins.2020.00269 (PMC7158873; doi:10.3389/fnins.2020.00269)
Supplement: Supplementary file 1 [file Table_1.docx]

Supplementary Material

# Supplementary Figures and Tables

*Supplementary Table 1 – Full clinical scores for all patients as well as overlap of overlap between pre-surgical tractography of the optic radiations and post-surgical structural scan. R = Right, L = Left, F = Female, M = Male; HH = Homonymous Hemianopia, HSQ = Homonymous Superior Quadrantanopia. *confrontational, + supported with electrophysiological data.*

| **Patient** | **Pre-surgery visual field** | **Pos-surgery visual field** | **Overlap at Maximum Cross Section area (%)** |
| --- | --- | --- | --- |
| Patient01 | Normal | Not Tested | 0 |
| Patient02 | No Co-operation | Not Tested | 0 |
| Patient03 | Normal*^+^ | Not Tested | 0 |
| Patient04 | Normal | R HSQ | 67 |
| Patient05 | Not Tested | Not Tested | 0 |
| Patient06 | Not Tested | Not Tested | 0 |
| Patient07 | Normal | Not Tested | 0 |
| Patient08 | Not Tested | Not Tested | 18 |
| Patient09 | Normal | Normal | 0 |
| Patient10 | No Co-operation | Not Tested | 0 |
| Patient11 | Normal* | Not Tested | 0 |
| Patient12 | Not Tested | R HSQ | 4 |
| Patient13 | Normal | R HSQ | 3 |
| Patient14 | R HH* | No Co-operation | 100 |
| Patient15 | R HH | R HH | 100 |
| Patient16 | Not Tested | Not Tested | 79 |
| Patient17 | Normal* | Normal* | 0 |
| Patient18 | Normal | L HH | 17 |
| Patient19 | Normal*^+^ | Not Tested | 29 |
| Patient20 | Normal*^+^ | Not Tested | 93 |
| Patient21 | Not Tested | Not Tested | 38 |
| Patient22 | Normal*^+^ | No Co-operation | 0 |
| Patient23 | Normal | Not Tested | 0 |
| Patient24 | Not Tested | Not Tested | 0 |
| Patient25 | Normal | Normal | 0 |
| Patient26 | Normal* | Not Tested | 0 |
| Patient27 | Not Tested | Not Tested | 5 |
| Patient28 | Not Tested | Not Tested | 7 |
| Patient29 | Not Tested | Not Tested | 75 |
| Patient30 | Normal* | Not Tested | 3 |
| Patient31 | Normal* | Normal *^+^ | 0 |
| Patient32 | Normal* | Normal* | 0 |
| Patient33 | Normal*^+^ | Not Tested | 0 |
| Patient34 | Not Tested | Not Tested | 49 |
| Patient35 | Normal | Not Tested | 0 |
| Patient36 | Not Tested | Not Tested | 0 |
| Patient37 | Normal*^+^ | Not Tested | 0 |
| Patient38 | Normal | Not Tested | 31 |
| Patient39 | Normal* | R HH* | 31 |
| Patient40 | Normal | Not Tested | 0 |
| Patient41 | R HSQ*^+^ | Not Tested | 0 |
| Patient42 | Normal*^+^ | L HH* | 46 |
| Patient43 | Normal | R HH | 63 |
